# Glucose-6-Phosphate Dehydrogenase Enhances Antiviral Response through Downregulation of NADPH Sensor HSCARG and Upregulation of NF-κB Signaling

**DOI:** 10.3390/v7122966

**Published:** 2015-12-17

**Authors:** Yi-Hsuan Wu, Daniel Tsun-Yee Chiu, Hsin-Ru Lin, Hsiang-Yu Tang, Mei-Ling Cheng, Hung-Yao Ho

**Affiliations:** 1 Department of Medical Biotechnology and Laboratory Science, College of Medicine, Chang Gung University, Tao-yuan 333, Taiwan; yihsuanwu@mail.cgu.edu.tw (Y.-H.W.); dtychiu@mail.cgu.edu.tw (D.T.-Y.C.); chengm@mail.cgu.edu.tw (M.-L.C.); 2 Healthy Aging Research Center, Chang Gung University, Tao-yuan 333, Taiwan; tangshyu@gmail.com; 3 Department of Laboratory Medicine, Chang Gung Memorial Hospital, Lin-Kou 333, Taiwan; 4 Molecular Medicine Research Center, Chang Gung University, Tao-yuan 333, Taiwan; rebecca19852006@hotmail.com; 5 Department of Biomedical Sciences, College of Medicine, Chang Gung University, Tao-yuan 333, Taiwan

**Keywords:** G6PD, NADPH, coronavirus, enterovirus, antiviral response, HSCARG

## Abstract

Glucose-6-phosphate dehydrogenase **(**G6PD)-deficient cells are highly susceptible to viral infection. This study examined the mechanism underlying this phenomenon by measuring the expression of antiviral genes—tumor necrosis factor alpha (*TNF-*α) and GTPase myxovirus resistance 1 (*MX1*)—in *G6PD*-knockdown cells upon human coronavirus 229E (HCoV-229E) and enterovirus 71 (EV71) infection. Molecular analysis revealed that the promoter activities of *TNF-*α and *MX1* were downregulated in *G6PD*-knockdown cells, and that the IκB degradation and DNA binding activity of NF-κB were decreased. The HSCARG protein, a nicotinamide adenine dinucleotide phosphate (NADPH) sensor and negative regulator of NF-κB, was upregulated in *G6PD*-knockdown cells with decreased NADPH/NADP^+^ ratio. Treatment of *G6PD*-knockdown cells with siRNA against *HSCARG* enhanced the DNA binding activity of NF-κB and the expression of *TNF-*α and *MX1*, but suppressed the expression of viral genes; however, the overexpression of HSCARG inhibited the antiviral response. Exogenous G6PD or IDH1 expression inhibited the expression of *HSCARG*, resulting in increased expression of *TNF-*α and *MX1* and reduced viral gene expression upon virus infection. Our findings suggest that the increased susceptibility of the *G6PD*-knockdown cells to viral infection was due to impaired NF-κB signaling and antiviral response mediated by HSCARG.

## 1. Introduction

Glucose-6-phosphate dehydrogenase (G6PD) deficiency is the most common enzymopathy in humans, affecting 400 million people worldwide [1]. G6PD plays an essential role in the pentose phosphate shunt for reducing nicotinamide adenine dinucleotide phosphate (NADP^+^) to NADPH. NADPH primarily serves to reduce equivalents for numerous biochemical reactions such as reductive biosynthesis, glutathione reduction, detoxification, and NADPH oxidase-mediated superoxide production. Therefore, G6PD helps to maintain cellular redox homeostasis [2,3], whereas G6PD deficiency predisposes cells to increased oxidative stress. *G6PD*-knockdown cells exhibit premature senescence, growth retardation, and increased susceptibility to stress-induced apoptosis [4,5,6]. Clinically, in addition to the classical association with hemolytic anemia [7,8], patients with G6PD-deficiency have an increased risk of degenerative diseases [9,10,11,12]. G6PD-deficient cells are more susceptible to enterovirus, coronavirus, and dengue virus infections [13,14,15]. These findings suggest that the G6PD status, and hence the redox environment, is a risk factor for viral infection. The mechanism underlying the effect of the redox environment on viral replication remains elusive.

Viral replication and spread is inhibited by the antiviral defense mechanisms of the host. The replication and spread normally involves activation of the antiviral innate immune responses and culminates in the production of type I interferons (IFNs) [16] and proinflammatory cytokines such as tumor necrosis factor alpha (TNF-α) [17,18]. Both IFNs and TNF-α are antiviral cytokines and display strong antiviral activity in host innate immunity [19]. Type I IFNs are the principal antiviral cytokines produced during the innate immune responses to viral infections, and upregulate antiviral proteins [20,21,22]. More than 300 IFN-stimulated genes (ISGs), which are initiated by type I IFN signaling, have been discovered. Some ISG-encoded proteins, such as GTPase myxovirus resistance 1 (MX1) [23], protein kinase R (PKR) [24], and 2′-5′-oligoadenylate synthetase (OAS) [25], are implicated in the antiviral state. Several ISGs are upregulated by infection with coronavirus or enterovirus [26,27,28]. TNF-α inhibited viral infections [19], and endogenous TNF-α inhibited the enhanced susceptibility to infectious diseases [29].

Reactive oxygen species (ROS) play crucial roles in many cellular processes including cell proliferation, differentiation, apoptosis, and signal transduction [30,31,32]. ROS have also been shown to trigger the signaling process of innate immune responses [33,34]. Oxidative stress affects viral replication partly by altering host immunity [35,36]. The inhibitory effect of the G6PD status on viral replication is unknown, which can be attributed to the alteration of the host innate immune response from modulating the redox homeostasis of the host cells.

HSCARG, also called NMRAL1, is an NADPH sensor; changes in the NADPH/NADP^+^ ratio can induce allosteric change and the subcellular redistribution of HSCARG [37,38]. HSCARG regulates the proteolysis of RelA and the phosphorylation of IKKβ [39] and plays an essential role in NF-κB signaling [39,40,41]. Moreover, HSCARG suppresses the TNF-α-stimulated activation of NF-κB [40], suggesting that the NADPH/NADP^+^ ratio or the redox status affects the NF-κB-mediated immune response through the modulation of HSCARG. HSCARG can inhibit TRAF3 ubiquitination and negatively regulate the cellular antiviral response [42]. Because of the crucial role of G6PD in maintaining cellular NADPH/NADP^+^, examining the relationship among the G6PD status, HSCARG, and host innate immune response is worthwhile.

This study addresses how the G6PD status affects the innate immune response to viral infection. The expression of viral genes is substantially higher in *G6PD*-knockdown cells infected with human coronavirus strain 229E (HCoV-229E) and EV71 than in infected control cells. The expression of antiviral genes, such as *MX1* and *TNF-*α, is upregulated, albeit to lower levels, in G6PD deficient cells. HSCARG, whose expression is enhanced in G6PD-deficient cells, inhibits IκB degradation and the DNA binding activity of NF-κB. Exogenous G6PD or isocitrate dehydrogenase 1 (IDH1), which increases cellular NADPH/NADP^+^, restores NF-κB-mediated antiviral response. These findings demonstrate that G6PD activity, and hence NADPH/NADP^+^ status, can affect antiviral immunity through the modulation of HSCARG and the NF-κB signaling cascade.

## 2. Materials and Methods

### 2.1. Materials

The following antibodies were used: anti-G6PD (Genesis Biotech, Taiwan**)**, anti-His tag, anti-IκB, anti-IDH1, anti-β-actin, anti-HSCARG, antihorseradish peroxidase (HRP)-conjugated antimouse IgG, and antirabbit IgG (Santa Cruz Biotechnology, Santa Cruz, CA, USA). TNF-α siRNA, HSCARG siRNA, and nontargeting siRNA were purchased from Dharmacon RNA Technologies (Lafayette, CO, USA). The ELISA kit for TNF-α was purchased from R & D systems (Minneapolis, MN, USA). BAY 11-7085 and all other chemicals were purchased from Sigma-Aldrich (St. Louis, MO, USA).

### 2.2. Cell Culture

A549 cells (human alveolar epithelial cell carcinoma), MRC-5 cells (human lung fibroblasts), and RD cells (human rhabdomyosarcoma) were purchased from the American Type Culture Collection (ATCC) (Manassas, VA, USA). The cells were cultured in Dulbecco modified Eagle medium supplemented with 10% fetal bovine serum and antibiotics (100 U/mL of penicillin and 100 μg/mL of streptomycin) at 37 °C in a humidified 5% CO_2_ atmosphere. The cassette for expressing G6PD and scrambled control (Sc) shRNA has been described previously [6]. The cassette was subcloned in pSuperior (Oligoengine, Seattle, WA, USA). The retroviral vectors were packaged into amphotropic virus by using PT67 cells, as previously prescribed [6]. The A549 cells were transduced with the packaged virus and selected for stable transfectants in a medium containing puromycin (2 μg/mL).

### 2.3. G6PD Activity

G6PD activity was measured spectrophotometrically at 340 nm according to the reduction of NADP^+^ in the presence of glucose-6-phosphate, as previously described [43]. In brief, the cells were collected and resuspended in lysis buffer (50 mM Tris-HCl (pH 7.4), 1% Triton X-100, 0.05% SDS, 150 mM NaCl, 1 mM EGTA, and 1 mM NaF). Cell lysates were centrifuged, and the supernatant was used in the assay. G6PD activity was analyzed by combining the protein and assay buffer (50 mM Tris-HCl (pH 8), 50 mM MgCl_2_, 4 mM NADP^+^, and 4 mM glucose 6-phosphate).

### 2.4. Virus Preparation and Plaque Assay

HCoV-229E was provided by Lai MM (Academia Sinica, Taiwan). The strain was propagated in the MRC-5 cells and purified through centrifugation. The lung carcinoma cell line A549 was used for the plaque assay. The viral titer was calculated according to the plaque formation on the A549 cells, as described previously [14]. Human enterovirus 71 (BrCr strain) was purchased from the ATCC (VR784). The virus was propagated, and plaque formation was assayed on the RD cells. The virus was aliquoted, quick-frozen on dry ice, and stored at −70 °C until used.

### 2.5. Quantitative-PCR

Total RNA was isolated using Trizol reagent. cDNA was performed using the SuperScript III system (Invitrogen, Carlsbad, CA, USA). Primers were designed according to the sequences of human antiviral gene cDNAs and those of viral genes. The sequences of the primers used in RT-PCR are listed in Table S1. Quantitative-PCR was performed using the IQ™ SyBr Green Supermix kit on an IQ5 Real Time Thermal Cycler (Bio-Rad, Hercules, CA, USA). Relative fold expression values were determined using the ΔΔ*C*t method.

### 2.6. Preparation of Cell Extracts and Western Blot Analysis

The cells were washed with ice-cold phosphate-buffered saline (PBS), scraped in lysis buffer (20 mM Tris-HCl (pH 7.5), 150 mM NaCl, 1% Nonidet P-40, 1 mM EDTA, 10 μg/mL aprotinin, 10 μg/mL leupeptin, 1 mM phenlmethylsulfonyl fluoride), and centrifuged at 40,000× *g* for 30 min at 4 °C to yield the whole-cell extract. Samples were denatured, electrophoresed on 10% SDS-polyacrylamide gel, and transferred to polyvinylidene difluoride (PVDF) membranes. The membranes were incubated overnight at 4 °C with an appropriate dilution of a primary antibody (1:1000) in Tris-buffered saline (TBS) (50 mM Tris-HCl, 150 mM NaCl, 0.05% (*w*/*v*) Tween-20 (pH 7.4)) containing 5% (*w*/*v*) bovine serum albumin (BSA). Membranes were incubated with a 1:4000 dilution of antirabbit or antimouse-HRP antibody for 1.5 h. The immunoreactive bands were visualized by ECL reagents (GE Healthcare, Little Chalfont, Buckinghamshire, UK), with the signals captured by exposure to X-ray film.

### 2.7. Plasmid Construction

The cDNA encoding His-tagged G6PD (Accession No. NM_000402), IDH1 (Accession No. NM_005896), and HSCARG (Accession No. NM_001305141.1) were cloned in pCI-neo plasmid (Invitrogen). The promoter region of the *TNF-α* and *MX1* gene was cloned from human genomic DNA, and subcloned in a pGL3-basic vector (Promega, Mannhein, Germany). A *MX1* promoter region devoid of NF-κB binding site (−244 to −235) was engineered. Mutant (−242 to −239; MX1P-mut; MX1M) and deletion (−244 to −235; MX1P-del; MX1D) forms of *MX1* promoter were established by site-directed mutagenesis (Stratagene, Amsterdam, Netherlands).

### 2.8. Transfection of Plasmids or siRNAs

The A549 cells (5 × 10^5^) were seeded on six-well plates and transfected 24 h later with plasmids using Lipofectamine 2000 (Invitrogen). During transient transfection with siRNA, the cells were transfected with 10 nM TNF-α or HSCARG siRNA. The nontargeting siRNA was used as a control for nonspecific effects of transfected siRNA. During transient transfection with plasmid, the cells were transfected according to the standard protocol (Invitrogen, CA, USA). The cells were harvested for analysis, or infected with HCoV-229E, 24 h after transfection.

### 2.9. Electrophoretic Mobility Shift Assay

*G6PD*-knockdown (Gi) and Sc A549 cells were infected with HCoV-229E for the indicated periods. Nuclear proteins were extracted using the Nuclear Extract kit (Active Motif, Carlsbad, CA, USA). Electrophoretic mobility shift assays (EMSAs) were carried out using the LightShift Chemiluminescent EMSA kit according to manufacturer protocol (Thermo Scientific, Rockford, IL, USA). In brief, the extract (10 μg) was incubated for 1 h at 4 °C with biotin end-labeled probe (Thermo Scientific, Rockford, IL, USA) containing NF-κB DNA-binding sites (5′-AGTTGAGGGGACTTTCCCAGGC-3′); the extract was resolved by nondenaturing PAGE on 4% polyacrylamide gel, transferred to nylon plus membrane, and determined by chemiluminescence according to manufacturer instructions.

### 2.10. Luciferase Assay

The A549 cells were transfected with 100 ng of reporter plasmid together with 400 ng of a pRL-null Renilla luciferase-encoding vector by using LF2000 (Invitrogen, Carlsbad, CA, USA). Twenty-four hours later, the A549 cells were infected with HCoV-229E at a multiplicity of infection (MOI) of 0.1. The cellular extract was assayed for luciferase activity using a dual luciferase assay and GLOMAX luminometer (Promega, Madison, WI, USA). Firefly luciferase activity was normalized to Renilla luciferase levels, and is expressed relative to pGL3-basic levels (RLU).

### 2.11. Determination of the NADPH/NADP^+^ Ratio

Determination of NADPH and NADP^+^ was performed as previously described [44]. In brief, the cells were washed twice with PBS and extracted in 80% methanol/10 mM KOH solution. After centrifugation at 14,000× *g* for 5 min, the supernatant was retained and completely dried under nitrogen gas. The sample was analyzed using ultra performance liquid chromatography (UPLC) equipped with a photodiode array detector. The sample was chromatographed on an Acquity HSST3 reversed-phase C18 column (2.1 mm × 150 mm, particle size of 1.8 mm; Waters Corp., Milford, MA, USA). The mobile phase was composed of 25 mM potassium monobasic phosphate buffer, pH 6 (solvent A), and 100% methanol (solvent B). The mobile phase conditions were as follows: solvent A, 2 min, gradient from 0 to 3%; solvent B, 0.5 min, gradient from 3% to 4%; solvent B, 2.5 min, gradient from 4% to 15%; solvent B, 2 min, gradient 15%; and solvent B, 1 min. The column temperature was maintained at 37 °C. The flow rate was set at 0.38 mL/min. Absorbance spectra were acquired over the wavelength range from 260 to 340 nm.

### 2.12. Statistical Analysis

Statistical analyses were carried out using a two-tailed Student’s *t* test. A *p* value of ≤0.05 was considered statistically significant. The data were representative of at least three independent experiments, and the values were given as the mean of replicate experiments ± standard deviation (SD).

## 3. Results

### 3.1. G6PD Deficiency Impairs the Expression of the Antiviral Genes, TNF-*α* and MX1, upon HCoV-229E or EV71 Infection

**Figure 1:**
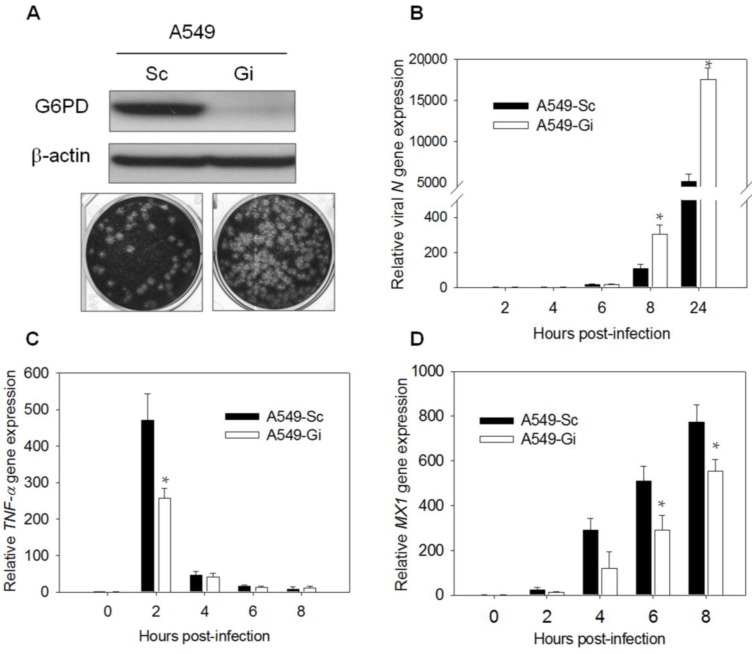
Expressions of antiviral gene *MX1* and *TNF-*α decrease upon HCoV 229E infection in A549-Gi cells. (**A**) A549-Sc and -Gi cells were harvested for determination of G6PD expression by western blotting. β-Actin was used as internal control. A549-Sc and -Gi cells were infected with HCoV-229E (0.1 MOI) for 24 h then viral particle was harvested and production was determined using plaque assay; (**B**) A549-Sc and -Gi cells were infected with HCoV-229E (0.1 MOI) for indicated time points. Viral *N* gene expression was determined by quantitative-PCR. Data were normalized to the value of infected A549-Sc cells at 2 h p.i.; (**C**) RNA was harvested from HCoV-229E-infected cells at indicated time p.i.. *TNF-*α gene expression was determined by quantitative-PCR. Data were normalized to the value of uninfected A549-Sc cells; (**D**) RNA was harvested from HCoV 229E-infected cells at indicated time points p.i.. *MX1* gene expression was determined by quantitative-PCR. Data were normalized to the value of uninfected A549-Sc cells. Values represent average ± SD of three experiments. * *p* < 0.05 as compared to A549-Sc cells.

The A549 cells were infected with a retroviral vector expressing G6PD-specific (Gi) and Sc shRNA. The generated A549-Gi and A549-Sc were used to delineate the mechanism underlying the increased susceptibility of G6PD-deficient cells to viral infection. The expression of G6PD was significantly reduced in A549-Gi cells compared with the A549-Sc cells (Figure 1A, top panel). The A549-Gi cells were infected with the HCoV-229E virus at a MOI of 0.1. The titer of progeny virus derived from the infected A549-Gi cells was significantly higher compared with the infected A549-Sc cells (Figure 1A, bottom panel). These findings are consistent with the temporal change in the expression of the viral *N* gene. The expression of the *N* gene increased with the time of infection (Figure 1B), and was higher in the A549-Gi cells than in the A549-Sc cells. The *N* gene level increased 304-fold in the A549-Gi cells versus an increase of 106-fold in the A549-Sc cells at 8 h postinfection (p.i.). At 24 h p.i., there was an over 17,000-fold increase in the *N* gene level in the A549-Gi cells and 5,000-fold increase in the A549-Sc cells. These findings are consistent with our previous findings [14].

The discrepancy between the viral replication in normal cells and G6PD-deficient cells correlates with their antiviral gene expression. Numerous antiviral genes were found to be upregulated in the A549 and MRC-5 cells after infection with HCoV-229E (Table 1). The expression of the antiviral genes *TNF-*α and *MX1* was studied in the infected A549-Gi and A549-Sc cells. *TNF-*α mRNA levels increased more than 400-fold in the A549-Sc cells at 2 h p.i., and returned to the original levels at 8 h p.i. (Figure 1C). The induction level was considerably lower in the A549-Gi cells, with the mRNA levels showing only a 250-fold increase at 2 h p.i. (Figure 1C, white bars). Likewise, the expression of the *MX1* gene increased during the infection course of HCoV-229E, and was significantly higher in the A549-Sc cells than in the A549-Gi cells (Figure 1D). The level of *MX1* mRNA increased over 22.8-fold at 2 h p.i. and 774.2-fold at 8 h p.i in the A549-Sc cells (Figure 1D). However, the level of induction was also reduced by 40% at 2 h p.i. and by 28% at 8 h p.i in the A549-Gi cells. The difference in inducibility of antiviral genes in control versus G6PD-deficient cells was observed in the case of EV71 infection, which increased *TNF-*α and *MX1* expression in RD cells (Table S2 and Figure S1). The expression of *TNF-*α (Figure S1B) and *MX1* (Figure S1C) genes in *G6PD-*knockdown RD-Gi cells was significantly lower than in RD-Sc cells. Furthermore, *G6PD*-knockdown increased the susceptibility to EV71 infection (Figure S1A, right panel; G6PD protein expression was shown in the left panel). These findings suggest that the expression of *TNF-*α and *MX1* is suppressed in G6PD-deficient cells.

### 3.2. TNF-*α* Knockdown Enhances Viral Replication in A549 Cells

**Figure 2:**
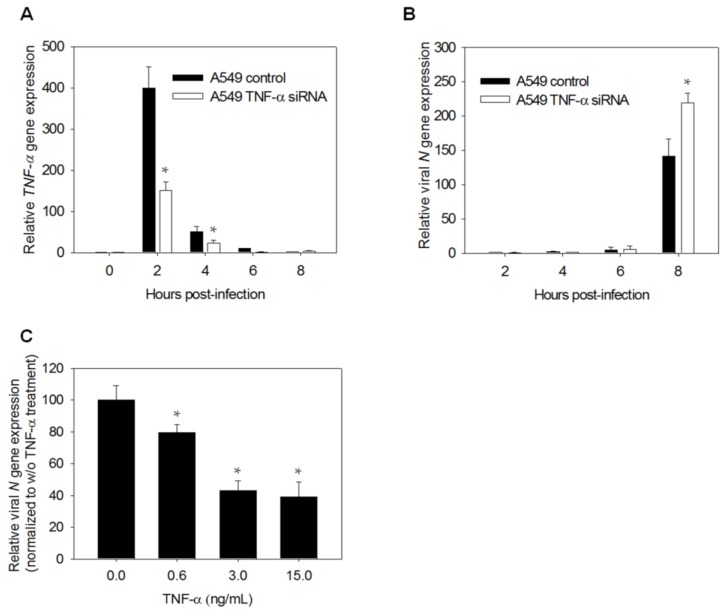
TNF-α inhibits viral replication in A549 cells. (**A**,**B**) The A549 cells were treated with control or TNF-α siRNA for 24 h and challenged with HCoV 229E of 0.1 MOI. Total RNA was harvested at indicated time and analyzed for *TNF-*α mRNA and viral *N* gene expression; (**C**) The A549 cells were challenged with different concentration of TNF-á for 24 h then infected with HCoV 229E of 0.1 MOI for 8 h. Total RNA was harvested for analyzing viral *N* gene expression by quantitative-PCR. Values represent average ± SD of three measurements. * *p* < 0.05, as compared to control siRNA-treated cells.

TNF-α is implicated in the modulation of the viral life cycle and the regulation of antiviral gene expression [17]. To test whether TNF-α expression limits virus replication, we knocked down TNF-α expression in A549 cells and examined their viral susceptibility. Pretreatment of the A549 cells with siRNA against *TNF-*α significantly reduced HCoV-229E-induced *TNF-*α expression throughout the course of infection (Figure 2A). Moreover, there was an increase in viral gene expression (Figure 2B). Conversely, TNF-α pretreatment significantly inhibited viral replication in a dose dependent manner (Figure 2C). These findings suggest that TNF-α plays a determinant role in the antiviral response. The reduced inducibility in G6PD-deficient cells may account for the increase in the viral replication of these cells.

**Table 1:** Time course pattern of antiviral gene expression in the A549 and MRC-5 cells upon HCoV 229E infection.

Cell Type	Gene	Fold Increase						
		0 h	2 h	4 h	6 h	8 h	10 h	24 h
A549	HCoV 229E *N* gene	N.D.	1	5.08 ± 1.30	20.92 ± 6.45	102.73 ± 15.24	946.55 ± 83.16	5566.24 ± 312.68
	*TNF-*α	1	432.58 ± 23.35	71.32 ± 0.41	27.05 ± 0.89	8.39 ± 1.29	7.71 ± 0.75	236.52 ± 33.27
	*IFN-*α	1	0.89 ± 0.11	1.76 ± 0.83	1.29 ± 0.43	2.30 ± 1.14	1.90 ± 0.87	5.27 ± 1.73
	*IFN-β*	1	1.31 ± 0.09	1.17 ± 0.15	1.43 ± 0.27	0.86 ± 0.29	1.81 ±0.35	504.07 ± 42.68
	*OAS*	1	1.77 ± 0.32	5.49 ± 0.22	9.73 ± 0.42	11.64 ± 2.53	15.16 ± 2.15	27.1 ± 1.25
	*PKR*	1	8.99 ± 2.94	7.45 ± 1.48	7.96 ± 1.51	8.08 ± 1.46	7.47 ± 1.54	8.72 ± 1.97
	*MX1*	1	123.69 ± 1.09	522.69 ± 50.95	736.82 ± 194.12	1390.95 ± 65.63	2381.91 ± 205.05	3709.11 ± 172.71
MRC-5	HCoV 229E *N* gene	N.D.	1	1.90 ± 0.12	6.96 ± 1.41	77.96 ± 14.32	311.13 ± 30.21	1145.40 ± 104.50
	*TNF-*α	1	1441.36 ± 343.85	975.38 ± 63.78	303.71 ± 60.77	137.94 ± 24.09	35.49 ± 2.29	7955.68 ± 664.50
	*IFN-*α	1	0.78 ± 0.20	0.93 ± 0.32	1.39 ± 0.54	0.79 ± 0.52	1.63 ± 0.24	0.88 ± 0.43
	*IFN-β*	1	0.92 ± 0.02	0.89 ± 0.12	0.98 ± 0.29	0.84 ± 0.05	1.23 ± 0.92	1194.91 ± 36.47
	*OAS*	1	11.27 ± 1.33	93.33 ± 0.41	289.97 ± 27.38	521.57 ± 170.79	997.31 ± 182.12	4775.85 ± 620.45
	*PKR*	1	4.10 ± 1.84	4.18 ± 1.69	5.16 ± 2.13	4.33 ± 0.24	5.00 ± 1.45	5.07 ± 0.51
	*MX1*	1	121.73 ± 44.20	512.70 ± 4.42	1345.64 ±105.59	1774.51 ± 185.55	1966.26 ± 333.56	6117.88 ± 674.95

N. D.: Not Detected.

### 3.3. Transcription of the TNF-*α* and MX1 Genes Is Reduced in G6PD-Knockdown Cells Upon Virus Infection

**Figure 3:**
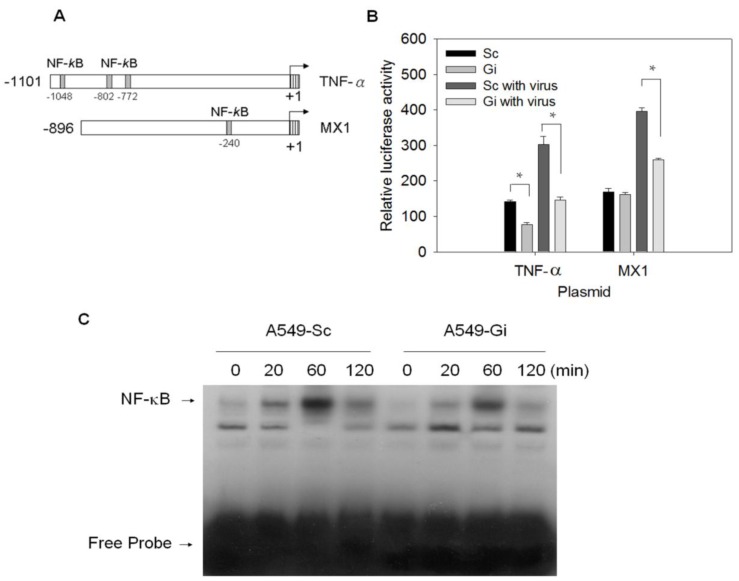
Promoter activities of antiviral genes, *TNF-*α and *MX1*, and NF-κB binding activity is highly correlated with G6PD status upon HCoV 229E infection. (**A**) Schematic presentation of the TNF-α (−1 to −1101) and MX1 (−1 to −896) promoter region are shown. The transcriptional start site is marked with an arrow (+1). NF-κB binding sites are indicated with labeled boxes; (**B**) A549-Sc and -Gi cells were transfected with control, TNF-α and MX1 reporter plasmid for 24 h, and were untreated or infected with HCoV-229E at MOI of 0.1. Luciferase activity was analyzed at 24 h p. i., and expressed as mean ± SD (*n* = 3) of fold change relative to vector control. * p < 0.05 as A549-Gi cells compared to A549-Sc cells alone or infected with virus, respectively; (**C**) A549-Sc and -Gi cells were infected with HCoV-229E at MOI of 0.1 for indicated periods. Nuclear extracts were prepared, and DNA binding activity was assayed by EMSA. DNA-protein complexes were resolved by eletrophoresis and detected by chemiluminescence.

The effect of the G6PD status on the expression of the *TNF-*α and *MX1* genes may be due to differences between the transcriptional levels of *G6PD*-knockdown and control cells. To test this hypothesis, A549-Sc and A549-Gi cells were transfected with reporter constructs, which have promoters of the *TNF-*α and *MX1* genes (Figure 3A). The transfected cells were assayed for luciferase activity after infection with HCoV-229E. The *TNF-*α and *MX1* promoter activity in the A549-Sc cells increased significantly at 24 h p.i. compared with that of the mock-infected control (Figure 3B). The promoter activities of the *TNF-*α and *MX1* genes were reduced in the A549-Gi cells. Moreover, lower promoter activities, especially those of the *TNF-*α gene, were observed in the A549-Gi cells compared with the A549-Sc cells under basal conditions. These findings suggest that the transcriptional activities of antiviral genes are affected by the G6PD status.

### 3.4. Binding Activity of NF-*κ*B Is Diminished in Virus-Infected G6PD-Knockdown Cells

Because the NF-κB binding sites were found in *TNF-*α [45] and *MX1* [46] promoter sequences (Figure 3A), G6PD deficiency probably inhibited the expression of these genes by altering the NF-κB signaling. The binding activity of NF-κB increased in the A549-Sc cells upon HCoV-229E infection, reaching the maximal level at 60 min p.i. and decreasing afterwards. The binding activity of NF-κB was higher in the A549-Sc cells than in the A549-Gi cells (Figure 3C). These findings suggest that G6PD deficiency leads to decreased NF-κB activation in virus-infected host cells.

To further confirm the role of NF-κB in the virus-induced upregulation of the *TNF-*α and *MX1* genes, we treated A549 cells with BAY 11-7085, a specific NF-κB inhibitor, and examined the temporal change in the expression of antiviral genes after infection. BAY 11-7085 significantly reduced the induction of the *TNF-*α and *MX1* genes (Figure S2). These data suggest that NF-κB activation is essential to virus-induced antiviral gene expression.

**Figure 4:**
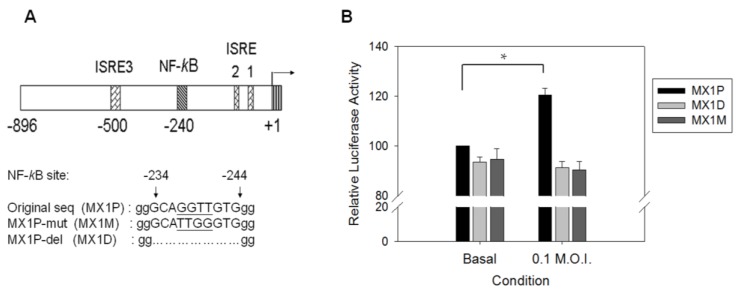
The promoter activity of *MX1* gene can be modulated by NF-κB in HCoV 229E-infected cells. (**A**) Schematic presentation of the *MX1* promoter region (−1 to −896). The transcriptional start site is marked with an arrow (+1). Three ISREs and one NF-κB (−234 to −244) binding site are indicated with labeled boxes. A *MX1* promoter region devoid of NF-κB binding site (−244 to −235) was engineered as described in Materials and Methods. The underlined letters indicates the nucleotides to be changed in MX1M-mut, and the dotted line indicates the nucleotides deleted in MX1P-del; (**B**) A549 cells were transfected with firefly and Renilla luciferase reporter plasmids containing MX1P, MX1D, or MX1M sequence. Twenty-four hours after transfection, cells were infected with HCoV 229E of 0.1 MOI. Luciferase activities was assayed at 48 h after transfection, and firefly luciferase activity was standardized by Renilla luciferase activity. The values are expressed relative to MX1P under basal condition. * *p* < 0.05 as compared to basal condition ones.

In contrast to the *TNF-*α gene that has the promoter endowed with NF-κB binding sites [45], little is known about the role of NF-κB in *MX1* promoter regulation. The modest increase in the promoter activity of the *MX1* gene raises doubt concerning the involvement of NF-κB in *MX1* promoter regulation. Various reporter vectors containing a luciferase gene linked to various forms of *MX1* gene promoter were constructed, and their activities were examined in the infected cells. The construct MX1P had the wild-type *MX1* gene promoter, while the constructs MX1D and MX1M had no binding sites or had mutant NF-κB binding sites (Figure 4A). HCoV-229E induced significant induction of the MX1P luciferase construct (Figure 4B). By contrast, the luciferase activity of MX1D and MX1M upon HCoV-229E infection was not significantly different from the uninfected control. These findings suggest that NF-κB plays a crucial role in the transcriptional regulation of the *MX1* gene.

### 3.5. HSCARG Expression Is Enhanced in G6PD-Knockdown Cells

**Figure 5:**
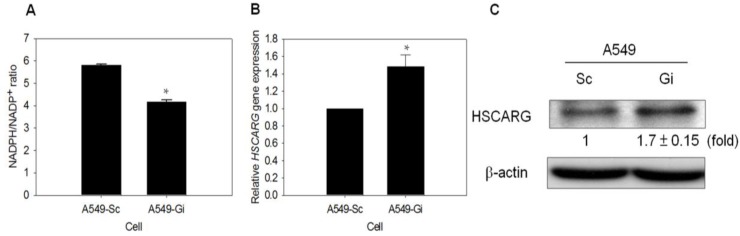
Expression of HSCARG increases in *G6PD*-knockdown A549 cells. (**A**) NADPH and NADP^+^ content were determined by UPLC, and NADPH/NADP^+^ ratio was calculated. * *p* < 0.05 as compared to A549-Sc cells; (**B**) The level of HSCARG mRNA was determined in A549-Sc and A549-Gi cells by quantitative-PCR. The level of *HSCARG* gene expression was normalized to that of A549-Sc cells. * *p* < 0.05, as compared to A549-Sc cells; (**C**) HSCARG protein in A549-Sc and A549-Gi cells was quantified by western blotting, and β-actin serves as internal control. The relative change in HSCARG protein level is shown as compared to A549-Sc cells. Numbers shown below the upper panel indicate the fold change in HSCARG expression relative to that of Sc cells.

HSCARG was proposed as a cellular redox sensor of the NADPH/NADP^+^ ratio, and a regulator of NF-κB signaling. HSCARG blocks IκB degradation and inhibits NF-κB activation [39]. The finding revealed a significantly lower NADPH/NADP^+^ ratio in A549-Gi cells compared with A549-Sc cells; this increases the possibility that HSCARG is involved in the regulation of NF-κB signaling (Figure 5A). The level of HSCARG transcript was elevated in the A549-Gi cells compared with that of the A549-Sc cells (Figure 5B). In addition, the level of HSCARG protein was higher in the A549-Gi cells than in the A549-Sc cells (Figure 5C). These results suggest that the G6PD status is involved in the regulation of HSCARG expression, thereby affecting NF-κB signaling and antiviral response.

### 3.6. Enhancement of the Antiviral Response and Inhibition of Viral Gene Expression Are Observed in HSCARG-Knockdown Cells

**Figure 6:**
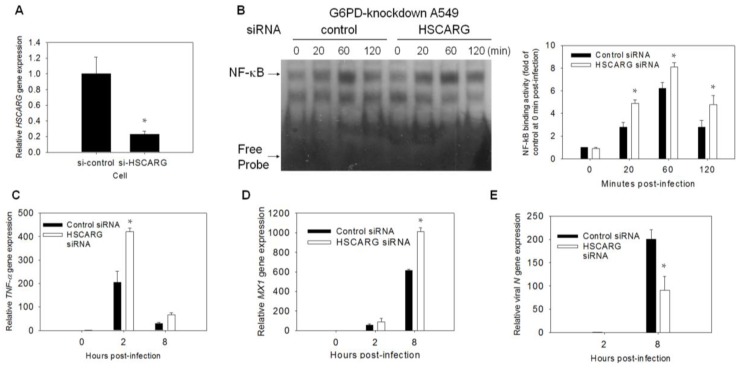
*HSCARG*-knockdown increases antiviral response and decreases viral replication. (**A**,**B**) A549-Gi cells were transfected with *HSCARG* siRNA. Total RNA was collected at 48 h after transfection, and *HSCARG* gene expression was determined by quantitative-PCR. Level of *HSCARG* was expressed relative to that of A549-Gi cells transfected with control-siRNA. For assay of NF-êB binding activity, A549-Gi cells were transfected with control or *HSCARG* siRNA for 48 h, and subsequently infected with HCoV 229E at MOI of 0.1. At indicated time points p.i., NF-êB binding activity was analyzed by EMSA. The temporal change in NF-êB binding activity is shown and quantified by densitometric scanning. The values represent average ± SD of five independent experiments; (**C**–**E**) A549-Gi cells were transfected with control or *HSCARG* siRNA for 48 h, and subsequently infected with HCoV 229E at an MOI of 0.1. Total RNA was harvested at indicated time points p.i., and analyzed for expression level of *TNF-*α (**C**), *MX1* (**D**), and viral *N* genes (**E**) by quantitative-PCR. Values represent average ± SD of three experiments. * *p* < 0.05 as compared to control siRNA treated cells.

To investigate the role of HSCARG in the antiviral response, A549-Gi cells were transfected with HSCARG-specific siRNA and the nontargeting siRNA control. Treatment of the A549-Gi cells with HSCARG-specific siRNA caused a significant reduction in the level of HSCARG mRNA (Figure 6A). The HSCARG-specific and nontargeting-siRNA-treated cells were infected with HCoV-229E (at a MOI of 0.1), and assayed for NF-κB binding activity. The NF-κB binding activity was enhanced in the HSCARG-specific, siRNA-treated cells. The NF-κB binding activity in these cells was compared with that of the nontargeting siRNA-treated cells (Figure 6B). The effect of the siRNA treatment on the temporal change of the expression of the *TNF-*α and *MX1* genes were studied in the infected cells. The levels of the transcripts of these genes were elevated in the HSCARG-specific, siRNA-treated cells compared with the nontargeting siRNA-treated cells during the course of HCoV-229E infection (Figure 6C,D). The elevation in the expression of the antiviral genes was accompanied by a converse reduction in the expression of the viral *N* gene (Figure 6E). The overexpression of HSCARG in the A549 cells decreased NF-κB binding activity. Therefore, antiviral gene expression increased coronaviral replication (Figure S3). These findings suggest that HSCARG acts diminish NF-κB activation, and suppresses antiviral gene expression in *G6PD*-knockdown cells.

### 3.7. Exogenous G6PD or IDH1 Expression Restores Antiviral Gene Expression and Inhibits Viral Replication

**Figure 7:**
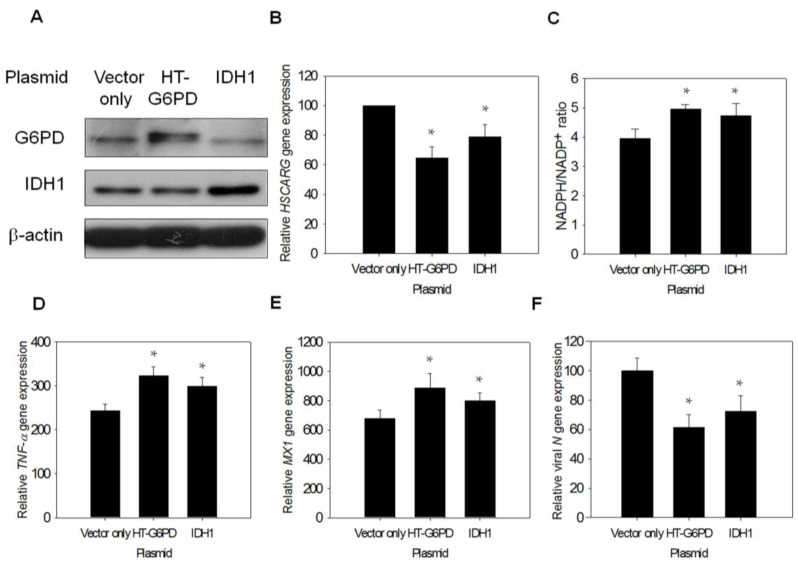
Expression of *G6PD* or *IDH1* gene augments antiviral gene expression in HCoV-229E-infected A549-Gi cells. (**A**,**B**) A549-Gi cells were transfected with expression construct encoding His-tagged G6PD or IDH1. Forty-eight hours later, RNA and cell lysate were harvested for analysis. Ectopic protein expression of HT-G6PD or IDH1 was measured by western blot. The level of *HSCARG* gene expression was determined by quantitative-PCR. The level of *HSCARG* gene expression was normalized to that of vector only-transfected cells; (**C**) NADPH and NADP^+^ content were determined by UPLC, and NADPH/NADP^+^ ratio was calculated. * *p* < 0.05 as compared to vector only control; (**D**–**F**) The A549 cells were transfected with expression construct encoding His-tagged G6PD or IDH1, and cells were infected with HCoV-229E at 0.1 MOI 48 h later; Total RNA was harvested for determination of expression level of *TNF-*α (at 2 h post-infection) (**D**); *MX1* (at 8 h post-infection) (**E**) and viral gene (at 8 h post-infection) (**F**) by quantitative-PCR. Data are expressed relative to that of cells transfected with control vector. Values present the average ± SD of three experiments. * *p* < 0.05 as compared to vector only-transfected cells.

As G6PD is indispensable in the maintenance of the redox status [47], it is possible that the alteration of cellular redox status changes HSCARG expression. To test this possibility, A549-Gi cells were transfected with constructs encoding His-tagged G6PD and IDH1. The expression of G6PD or IDH1 in the A549-Gi cells led to the diminished expression of the HSCARG transcript (Figure 7A,B), and significantly increased NADPH/NADP^+^ ratio compared with vector-transfected control cells (Figure 7C). Because knockdown of G6PD expression could suppress antiviral gene expression and enhance *N* gene expression, it would be worthwhile to investigate how exogenous G6PD or IDH1 expression could reverse such changes. Exogenous G6PD or IDH1 expression led to an increase in *TNF-*α and *MX1* gene expression upon HCoV-229E infection (Figure 7D,E). This was accompanied by decreased viral *N* gene expression (Figure 7F). These findings strongly support the notion that cellular G6PD activity and the NADPH/NADP^+^ status affect NF-êB signaling by modulating HSCARG expression.

## 4. Discussion

**Figure 8:**
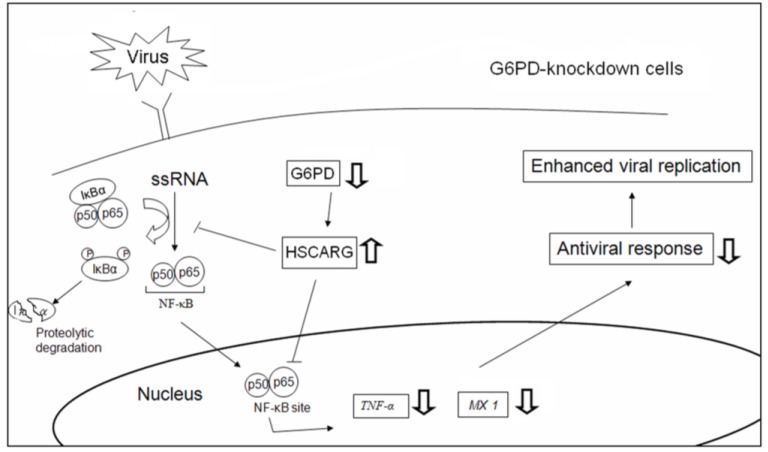
A schematic diagram shows how G6PD enhances antiviral response. In normal cells, viral infection induces IκB degradation and NF-κB translocation, which promote antiviral response and inhibit virus replication. In *G6PD*-knockdown cells, the fall in NADPH/NADP^+^ ratio leads to HSCARG upregulation, which negatively affect NF-κB signaling and antiviral gene expression in response to viral infection. In this manner, viral replication is enhanced. An upward arrow indicates increase in expression or response, while a downward arrow indicates decrease in expression or response.

This study demonstrates that cellular redox affects viral replication, at least partly, via regulation of the antiviral immune response. The G6PD status, a determinant of the NADPH/NADP^+^ ratio, affects cellular HSCARG expression, and modulates the level of NF-κB activation and the expression of antiviral genes in response to viral infection. Our findings support the notion that a change in the redox status can affect antiviral signaling (Figure 8).

Viruses, such as the influenza virus and the coronavirus, elicit cellular innate immunity [48,49,50,51,52]. We have previously shown that *G6PD*-knockdown cells are more susceptible to viral infection [13,14]. It is probable that the increased susceptibility to viral infections may be partly due to the impaired immune response of *G6PD*-knockdown cells. The expression of cytokine genes (such as *IFN-*α*/*β and *TNF-*α) and antiviral genes (such as IFN-stimulated genes (ISGs)) increases in A549 and MRC-5 cells upon HCoV-229E infection, and increases in RD cells upon EV71 infection. The expression of the *TNF-*α and *MX1* genes are differentially regulated in both the normal and G6PD-deficient cells, revealing the modulatory effect of cellular redox status on the innate immune system. Consistent with previous studies reporting that TNF-α exerts an antiviral effect [17,19,53], the present study demonstrates that TNF-α protects epithelial cells from HCoV-229E infection. Knockdown of TNF-α expression can enhance HCoV-229E replication, whereas TNF-α pretreatment can decrease the susceptibility of infection in *G6PD*-knockdown A549 cells. Moreover, the kinetics of *MX1* gene induction is of particular interest. The *MX1* gene is induced in cells upon infection with HCoV-229E or EV71 at a time point when the type I IFN level is low. These findings suggest that the induction of the *MX1* gene may occur via a type I IFN-independent pathway.

The transcriptional activation of TNF-α and antiviral genes in virus-infected cells are mediated through a complex signaling circuitry. The NF-κB signaling pathway represents an essential regulator of antiviral defense. NF-κB is a key transcription factor involved in the regulation of the immune response [54,55,56]. It is not unprecedented that viral infection is associated with NF-κB signaling, which may benefit the host or the invading microbe [57]. Infection of cells with HCoV-229E results in IκB degradation and increases NF-κB binding. This is accompanied by increased transcription of *TNF-*α and *MX1* genes, of which the promoters are endowed with NF-κB binding sites. The necessity of NF-κB in the activation of the antiviral response against HCoV-229E is demonstrated by the ability of BAY11-7085 to inhibit the *TNF-*α and *MX1* gene induction in infected cells.

NF-κB activation is sensitive to cellular redox changes; NF-κB is normally activated under oxidative stress [58]. Paradoxically, NF-κB activation is diminished in coronavirus-infected G6PD-deficient cells, suggesting the involvement of additional factors in the antiviral signaling pathway. HSCARG may act as a redox sensor, which functionally alters and undergoes differential translocation in response to different cellular NADPH levels [59]. HSCARG translocates from the cytoplasm to the nucleus under low NADPH conditions [38,39]. HSCARG has been implicated in the fine-tuning of NF-κB activation. Several studies have suggested that HSCARG is involved in the regulation of antiviral signaling. The HSCARG level increases in *G6PD*-knockdown cells. Treatment with *HSCARG* siRNA increases the antiviral response and reduces the viral replication in *G6PD*-knockdown cells. It is likely that G6PD deficiency impairs the cellular capacity to produce NADPH, thus enhancing HSCARG expression and its nuclear translocation. In addition, the exogenous expression of IDH1 is inhibitory to HSCARG expression. Therefore, it is conceivable that HSCARG suppresses NF-κB activity via modulation of IκB phosphorylation/degradation, leading to reduction in the expression of antiviral genes such as *TNF-*α and *MX1*. This reasoning may account in part for the increased susceptibility of cells to coronavirus or enterovirus. Our findings suggest that the redox status may regulate the antiviral response and viral replication via the HSCARG-NF-κB signaling pathway.

The clinical implications of how the G6PD status affects cellular susceptibility to viruses such as coronavirus [14], enterovirus [13], and dengue virus [15] remain to be determined. Certain clinical findings regarding individuals with G6PD-deficiency are compelling: They develop symptoms (e.g., hemolysis and pancreatitis) that are more severe after infection with the hepatitis E virus [60,61], they are more susceptible to dengue virus type 2 infection than normal subjects [15], and they develop severe pneumonia symptoms after microbial infection [62,63]. Redox imbalance may account for the impairment of antiviral immunity in patients with G6PD-deficiency. Consistent with this, interleukin-10 expression is dysregulated in monocytes from G6PD-deficient individuals [64].

The roles of G6PD in the maintenance of human health and development of disease have been extensively reviewed elsewhere [1]. Drug-induced cytotoxicity in G6PD-deficient cells is normally attributed to their reduced ability to regenerate NADPH and glutathione [4,44,65]. Deficit of NADPH can also lead to reduced NADPH oxidase activity and superoxide generation [66]. Increased superoxide generation by NADPH oxidases in diabetic mice is associated with increased G6PD activity [67]. The correlation between superoxide generation and G6PD activity has been observed in such pathophysiological situations as liver hypertrophy [68], and cardiovascular dysfunction [69]. Likewise, nitric oxide production can be affected [70]. However, the interplay between antioxidative (*i.e.*, NADPH production and maintenance of endogenous antioxidants) and pro-oxidative (*i.e.*, NADPH and superoxide production) roles of G6PD may be more complicated than previously thought. For instance, G6PD-deficient granulocytes have an impaired ability to generate superoxide, nitric oxide and hydrogen peroxide [71]. On the other hand, G6PD-deficient neutrophils show increased superoxide and hydrogen peroxide production [72]. More studies are needed to solve such a riddle.

This study demonstrates that the G6PD status modulates innate antiviral immunity via the HSCARG-NF-κB signaling axis. Therefore, it is likely that the G6PD status is a factor in determining the host-cell–microbe interaction.

## Supplementary Materials

Supplementary materials can be accessed at: http://www.mdpi.com/1999-4915/7/12/2966/s1.
