# Supplementary material for: Glucose-6-Phosphate Dehydrogenase Enhances Antiviral Response through Downregulation of NADPH Sensor HSCARG and Upregulation of NF-κB Signaling
Source: Viruses. 2015 Dec 17;7(12):6689–706. doi: 10.3390/v7122966 (PMC4690889; doi:10.3390/v7122966)
Supplement: Supplementary file 1 [file viruses-07-02966-s001.docx]

**Supplemental Information**

Glucose-6-Phosphate Dehydrogenase Enhances Antiviral Response through Downregulation of NADPH Sensor HSCARG and Upregulation of
NF-κB Signaling

Yi-Hsuan Wu, Daniel Tsun-Yee Chiu, Hsin-Ru Lin, Hsiang-Yu Tang, Mei-Ling Cheng
and Hung-Yao Ho


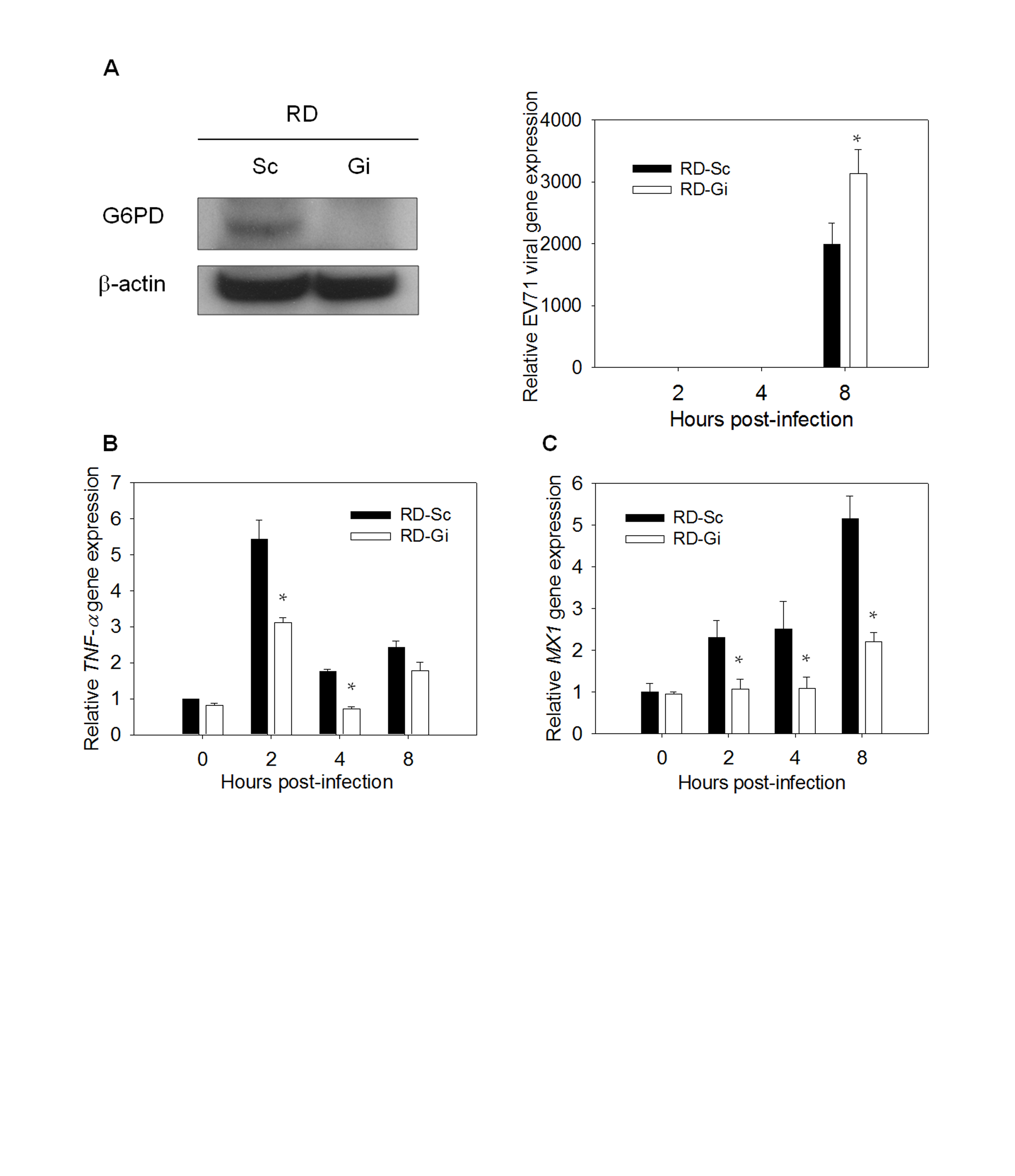


**Figure S1.** Expression of antiviral genes *MX1* and *TNF-α* decrease upon EV71 infection in RD-Gi cells. (**A**). RD-Sc and -Gi cells were harvested for determination of G6PD expression by western blot. β-Actin was used as loading control. RD-Sc and -Gi cells were infected with EV71 (MOI = 0.1) for indicated time points. EV71 viral gene expression was determined by quantitative-PCR. Data were normalized to the value of infected RD-Sc cells at 2 h p.i.. Values represent average ± SD of three experiments. * *p* < 0.05 as compared to RD-Sc cells. (**B**) RNA was harvested from EV71-infected cells at indicated time points p.i., and the level of *TNF-α* gene expression was determined by quantitative-PCR. Data were normalized to the value of uninfected RD-Sc cells. (**C**) RNA was harvested from HCoV-229E-infected cells at indicated time points p.i.. *MX1* gene expression was determined by quantitative-PCR at indicated time points. Data were normalized to the value of uninfected RD-Sc cells. Values represent average ± SD of three experiments. * *p* < 0.05 as compared to RD-Sc cells.


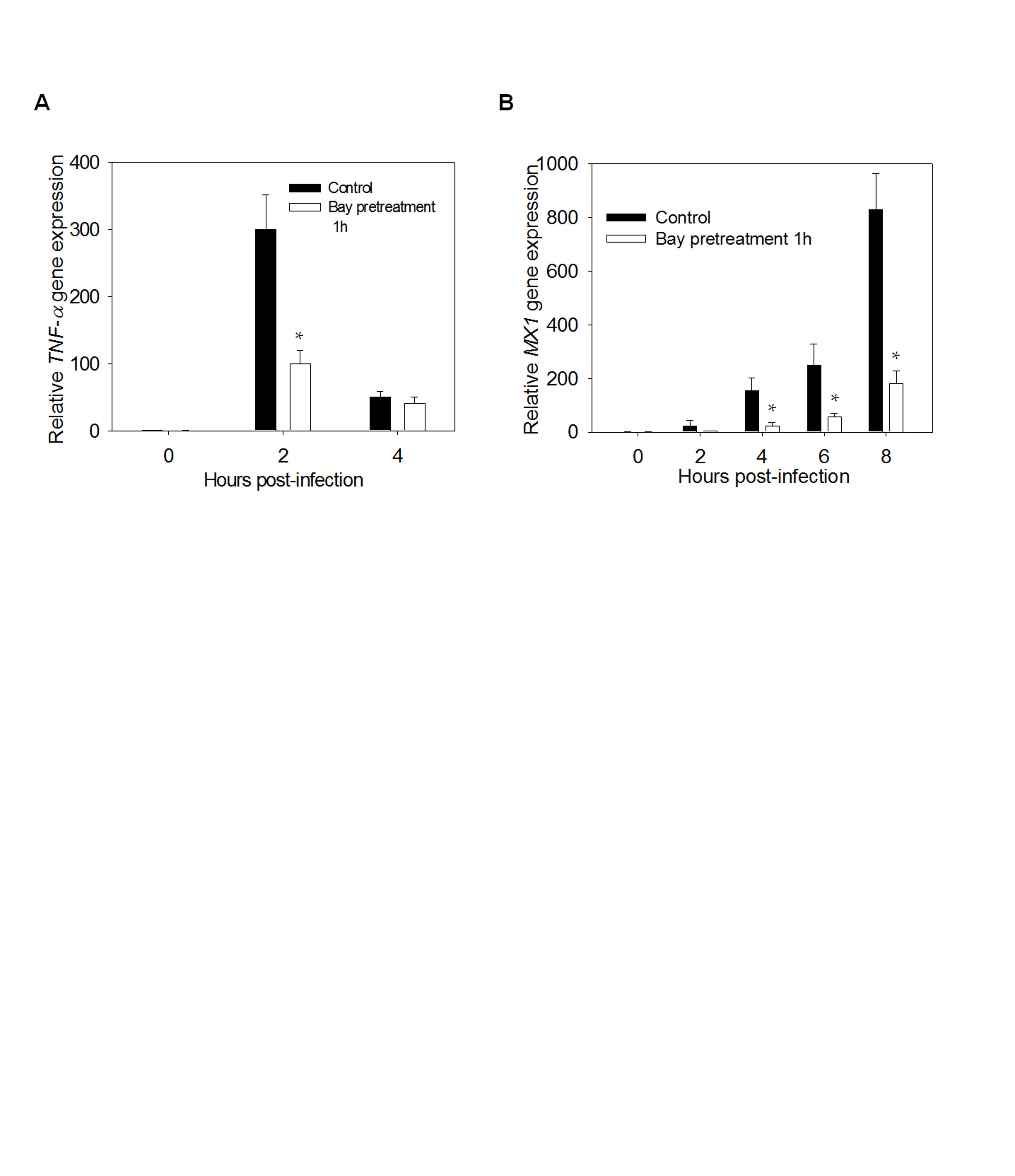


**Figure S2.** NF-κB is involved in the regulation of *TNF-α* and *MX1* gene expression upon HCoV-229E infection. (**A,B**) Cells were pretreated with BAY 11-7085 (10 μM) or DMSO (control) for 1 h, washed by PBS, and infected with HCoV-229E (0.1 MOI) for indicated time points. Total RNA was extracted and analyzed for *TNF-α* and *MX1* gene expression by quantitative-PCR. Average values ± SD of three measurements each of two independent experiments are given. * *p* < 0.05, as compared to DMSO-treated cells.


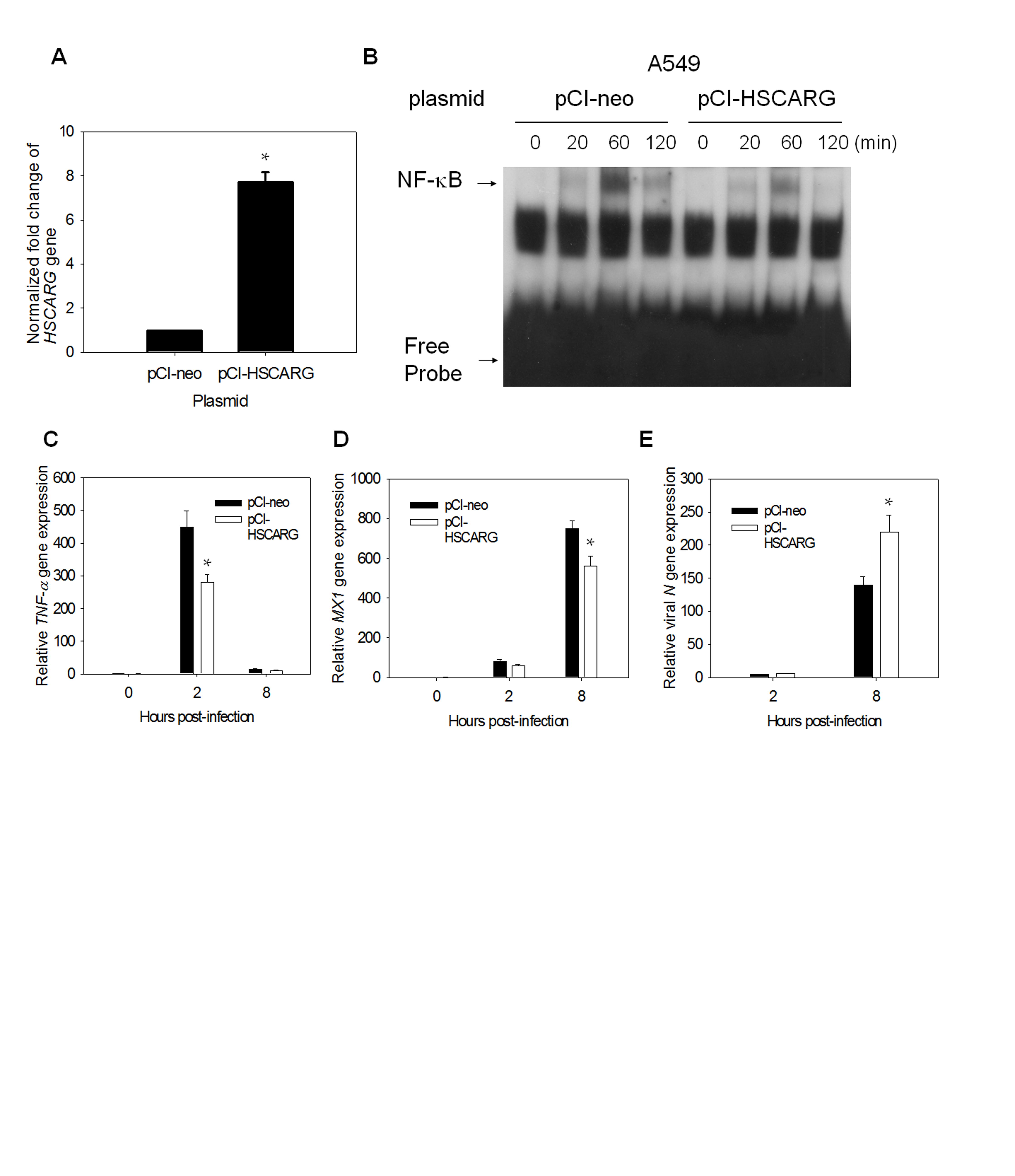


**Figure S3.** *HSCARG* overexpression decreases antiviral response and increases viral replication. (**A,B**) A549 cells were transfected with pCI-neo control vector or *HSCARG* expression vector. Total RNA was collected at 48 h after transfection, and *HSCARG* gene expression was determined by quantitative-PCR. Level of *HSCARG* is expressed relative to that of A549 cells transfected with control plasmid (pCI-neo). For assay of NF-κB binding activity, A549 cells were transfected with control vector or *HSCARG* expression vector plasmid for 48 h, and subsequently infected with HCoV-229E at MOI of 0.1. At indicated time points p.i., NF-κB binding activity was analyzed by EMSA. (**C-E**) A549 cells were transfected with control or *HSCARG* expression plasmids for 48 h, and subsequently infected with HCoV-229E at MOI of 0.1. Total RNA was harvested at indicated time points p.i., and analyzed for expression level of *TNF-α* (**C**), *MX1* (**D**) and viral *N* genes (**E**) by quantitative-PCR. Values represent average ± SD of three experiments. * *p* < 0.05 as compared to vector control.

**T**able S1 Sequences of primers for the amplification of selected genes evaluated by real-time PCR.

| **Gene** | **Sense (5’ to 3’)** | **Antisense (5’ to 3’)** | **Target size (bp)** |
| --- | --- | --- | --- |
| *ACT-β^a^* | TCCACCTTCCAGCAGATG | GTGTAACGCAACTAAGTCATAG | 108 |
| *MX1* | ACCACAGAGGCTCTCAGCAT | CTCAGCTGGTCCTGGATCTC | 200 |
| *OAS* | AGAAGGCAGCTCACGAAAC | GAAGCAGGAGGTCTCACCAG | 194 |
| *PKR* | GCCCCTTCCCAAGTAAAACT | GGCACTGTAAAATGGGTGCT | 201 |
| *IFN-α* | TCCATGAGATGATCCAGCAG | ATTTCTGCTCTGACAACCTCCC | 274 |
| *IFN-β* | GATTCATCTAGCACTGGCTGG | CTTCAGGTAATGCAGAATCC | 186 |
| *TNF-α* | CCTGTGAGGAGGACGAAC | CGAAGTGGTGGTCTTGTTG | 167 |
| *HSCARG* | AATACGTCGGCCAGAACATC | TCAGGGCATAGAAACGGAAC | 183 |
| HCoV-229E *N* gene | AGGCGCAAGAATTCAGAACCAGAG | AGCAGGACTCTGATTACGAGAAAG | 309 |
| EV71 viral gene | ACTGACCAAGGACACTTCAC | CCAGTGTGAGTTCCAAGTTT | 166 |

^a^ Housekeeping gene transcript serving as the normalization control for quantitative-PCR.

**T**able S2 Time course pattern of antiviral gene expression in RD cells upon EV71 infection.

| **Fold increase** | | | | | | |
| --- | --- | --- | --- | --- | --- | --- |
| **Cell type** | **Gene** | **0 hr** | **2 hr** | **4 hr** | **6 hr** | **8 hr** |
| RD | EV71 viral gene | N.D. | 1 | 1.00 ± 0.09 | 112.70 ± 1.70 | 2626.3 ± 361.4 |
|  | *TNF-α* | 1 | 5.44 ± 0.62 | 1.67 ± 0.24 | 2.58 ± 0.40 | 2.69 ± 0.52 |
|  | *IFN-α* | 1 | 5.04 ± 0.93 | 9.46 ± 3.25 | 3.03 ± 3.60 | 1.77 ± 0.09 |
|  | *IFN-β* | 1 | 0.52 ± 0.03 | 1.23 ± 0.11 | 2.94 ± 1.06 | 12.48 ± 1.02 |
|  | *OAS* | 1 | 0.82 ± 0.25 | 1.13 ± 0.06 | 1.20 ± 0.17 | 1.13 ± 0.22 |
|  | *PKR* | 1 | 3.39 ± 0.90 | 4.35 ± 1.71 | 3.14 ± 0.24 | 3.61 ± 0.59 |
|  | *MX1* | 1 | 1.84 ± 0.20 | 2.06 ± 0.27 | 3.13 ± 0.12 | 5.12 ± 0.42 |

N. D.: Not Detected.

© 2015 by the authors; licensee MDPI, Basel, Switzerland. This article is an open access article distributed under the terms and conditions of the Creative Commons by Attribution (CC-BY) license (http://creativecommons.org/licenses/by/4.0/).
